# Development and internal validation of machine learning algorithms to predict patient satisfaction after total hip arthroplasty

**DOI:** 10.1186/s42836-021-00087-3

**Published:** 2021-09-02

**Authors:** Siyuan Zhang, Jerry Yongqiang Chen, Hee Nee Pang, Ngai Nung Lo, Seng Jin Yeo, Ming Han Lincoln Liow

**Affiliations:** 1 Yong Loo Lin School of Medicine, National University of Singapore, 1E Kent Ridge Road, NUHS Tower Block, Level 11, Singapore, 119228 Singapore; 2 Department of Orthopaedic Surgery, Singapore General Hospital, 20 College Road, Academia, Level 4, Singapore, 169856 Singapore

**Keywords:** Machine learning, Artificial intelligence, Total hip arthroplasty, Satisfaction, Patient-reported outcome measures

## Abstract

**Background:**

Patient satisfaction is a unique and important measure of success after total hip arthroplasty (THA). Our study aimed to evaluate the use of machine learning (ML) algorithms to predict patient satisfaction after THA.

**Methods:**

Prospectively collected data of 1508 primary THAs performed between 2006 and 2018 were extracted from our joint replacement registry and split into training (80%) and test (20%) sets. Supervised ML algorithms (Random Forest, Extreme Gradient Boosting, Support Vector Machines, Logistic LASSO) were developed with the training set, using patient demographics, comorbidities and preoperative patient reported outcome measures (PROMs) (Short Form-36 [SF-36], physical component summary [PCS] and mental component summary [MCS], Western Ontario and McMaster’s Universities Osteoarthritis Index [WOMAC] and Oxford Hip Score [OHS]) to predict patient satisfaction at 2 years postoperatively. Predictive performance was evaluated using the independent test set.

**Results:**

Preoperative models demonstrated fair discriminative ability in predicting patient satisfaction, with the LASSO model achieving a maximum AUC of 0.76. Permutation importance revealed that the most important predictors of dissatisfaction were (1) patient’s age, (2) preoperative WOMAC, (3) number of comorbidities, (4) preoperative MCS, (5) previous lumbar spine surgery, and (6) low BMI (< 18.5).

**Conclusion:**

Machine learning algorithms demonstrated fair discriminative ability in predicting patient satisfaction after THA. We have identified modifiable and non-modifiable predictors of postoperative satisfaction which could enhance preoperative counselling and improve health optimization prior to THA.

## Introduction

Total hip arthroplasty (THA) is a commonly performed and effective operation for symptomatic hip osteoarthritis, leading to significant improvements in pain, function and quality of life [1]. To assess a patient’s response after THA, patient-reported outcome measures (PROMs) and patient satisfaction are often used. While PROMs predominantly measure improvements in pain and function, patient satisfaction is a unique and holistic outcome that reflects the individual’s subjective quality of life improvements in relation to preoperative expectations [2, 3].

This is especially important since the primary aim of elective joint arthroplasty is to improve the patient’s quality of life. Prior research has shown that patient satisfaction is a complex and multifactorial phenomenon, with postoperative satisfaction influenced by various determinants such as age, sex, mental health, preoperative expectations, as well as postoperative functional improvements [4–7]. Despite the efficacy of THA, recent literature has shown that around 10–20% of patients remain dissatisfied after surgery [3, 8–10].

As healthcare systems worldwide transition to a value-based, patient-centered model, patient satisfaction will become increasingly important as a measure of success in total joint arthroplasty (TJA) [11, 12]. Being able to predict patients who may be dissatisfied enables surgeons to provide individualized preoperative counselling which could help address unrealistic patient expectations regarding THA [13, 14]. This information may also allow for early intervention and optimization of the patient’s physical and mental health. To do so, accurate prediction models are required. One way such models can be developed is using machine learning (ML) algorithms. ML is a subset of artificial intelligence (AI) that uses computer algorithms capable of learning from real-world data and using these insights to predict an outcome without being explicitly programmed [15, 16]. In recent years, the use of ML and AI in medicine have gained traction due to their ability to accurately predict medical outcomes, ranging from heart failure to cancer prognosis [17–19]. In orthopedic surgery, prior studies by Fontana et al, Huber et al and Kunze et al have demonstrated the feasibility of ML algorithms in predicting PROM improvements after TJA [8, 9, 20]. More recently, Kunze et al and Farooq et al have also tried to predict patient satisfaction after TKA, achieving an AUC of 0.77 and 0.81 respectively [21, 22]. However, there have been no prior studies using ML algorithms to predict patient satisfaction after THA.

Thus, the primary aim of our study was to evaluate whether ML algorithms can predict patient satisfaction after THA. Our secondary aim was to identify the underlying variables which drive prediction in these models.

## Materials and methods

### Data

Following ethics approval by the institutional ethics review board (CIRB 2020/2843), we reviewed prospectively collected data of consecutive patients who underwent elective primary THA from a single institution’s joint replacement registry between 2006 and 2018. We identified 1996 adult patients who had undergone unilateral primary THA during this period, of which 1508 (75.6%) had completed their 2 year postoperative follow-up for patient satisfaction and PROMs (SF-36 PCS/MCS, WOMAC, OHS).

### Outcomes

Patient satisfaction was recorded at 2 years postoperatively and was rated on a 6-level Likert scale, similar to the approach described by Bourne et al [23]. Patients were asked to grade their level of satisfaction (“terrible”, “poor”, “fair”, “good”, “very good” or “excellent”) and this was dichotomized into patients who were satisfied (“excellent”, “very good” or “good”) and patients who were not satisfied (“terrible”, “poor” or “fair).

### Input variables

Input variables used in this study were prospectively collected by our joint replacement registry. These included patient demographics, comorbidities and PROMs (Table 1). PROMs collected include the Short Form-36 (SF-36), Western Ontario and McMasters Universities Osteoarthritis Index (WOMAC) and Oxford Hip Score (OHS).

**Table 1:** Input variables for ML models

Demographics	Comorbidities	Preoperative PROMs
Age* Sex BMI (numerical)* BMI (categorical)*	Number of comorbidities* Diabetes Hypertension High cholesterol Ischemic heart disease, Stroke Renal disease Back pain Depression Previous hip surgery* Previous knee surgery Previous lumbar spine surgery*	SF-36 PCS* SF-36 MCS* WOMAC* OHS*

*10 candidate variables selected from recursive feature elimination

The SF-36 is a generic health questionnaire that measures an individual’s health-related quality of life, and its 8 domains are commonly aggregated into the physical component summary (PCS) and mental component summary (MCS) [24]. On the other hand, the WOMAC is a disease-specific questionnaire for lower limb arthritis, with 24 items grouped into 3 dimensions of pain (5 items), stiffness (2 items) and physical function (17 items) [25]. The total WOMAC score was calculated by summing the aggregate scores for the 3 dimensions and transforming it to a scale of 0–100. Lastly, the OHS is a 12-item questionnaire that assesses hip function and pain. Each item has 5 response options, giving a score between 1 and 5. These scores are summated into a final score ranging between 12 and 60, with a higher score indicating greater disability [26].

### Data handling and model training

Data analysis and modelling were performed using Python 3.7 (Python Software Foundation, Wilmington, DE, USA), the Anaconda Distribution (Anaconda, Inc., Austin, TX, USA) and R software, version 4.0.3 (R Foundation for Statistical Computing, Vienna, Austria, 2019). We used four of the most popular supervised ML algorithms (Scikit-learn version 0.24): Random Forest (RF), Extreme Gradient Boosting (XGB), Support Vector Machine (SVM), and Logistic Regression with L1-regularization (LASSO).

The 1508 primary THAs were randomly split into a training set and testing set at an 80–20 ratio. Data in the training set (*n* = 1206) were used to train and fit the various ML algorithms while the independent test (*n* = 302) was reserved for the final evaluation of the model’s performance. The only missing input variable was body mass index (BMI) (*n* = 102, 6.8%). Missing BMI values in the training and test set were imputed using the mean BMI of the training and test sets respectively. Due to class imbalance of the outcomes (majority of patients were satisfied), which could adversely impact the predictive performance of some ML algorithms, we employed a technique called random oversampling during model training. Random oversampling is a simple upsampling technique that randomly resamples the minority class to reach balanced class ratios in the training set [27].

Recursive feature elimination (RFE) using an RF classifier was used to select 10 candidate variables from the larger pool of input variables to train the ML models. RFE selects candidate variables by iteratively calculating the importance scores for each variable and ranking them in order of importance. All ML models were trained using 5-fold stratified cross-validation in the training set to optimize their hyperparameters before final evaluation of their performance on the independent test set.

### Model evaluation

All our models were evaluated on an independent test set that was not involved in model training. The main evaluation metric used was the area under the receiver operating characteristic curve (AUC), which is a measure of the model’s ability to discriminate between two different classes. A perfect classifier would have an AUC of 1.0 while a completely random classifier (i.e. flipping a coin) would have an AUC of 0.5. Generally speaking, an AUC of 0.7–0.8 is fair, 0.8–0.9 is good and 0.9–1.0 is excellent. Apart from the AUC, other evaluation metrics used include the Brier score, sensitivity and specificity and calibration slope and intercept.

### Variable importance

The relative importance of input variables was assessed using permutation importance, a model-agnostic method that has been shown to generate reliable insights correlating with clinical intuition [28]. The permutation importance of a variable is defined as the decrease in accuracy (AUC in this case) of the trained model on the test set when the said variable is randomly shuffled, thus giving us an estimate of how much the input variable contributes to predictive performance.

## Results

### Baseline patient characteristics

Baseline patient characteristics of the 1508 THAs in our study are summarized in Table 2. 69.8% (*n* = 1052) of the patients were female and 30.2% (*n* = 456) were male. The mean age was 62.9 (SD:12.1) and mean BMI was 25.8 (SD:4.9).

**Table 2:** Baseline Patient Characteristics

Variables	Training set (*n* = 1206)	Test set (*n* = 302)	*P*-value
Age	62.8 (12.1)	63.2 (11.8)	0.623
Sex (Female)	841 (69.7%)	211 (69.9%)	0.964
BMI	25.9 (4.8)	25.3 (4.3)	0.075
BMI (categorical)			0.488
< 18.5	39 (3.2%)	14 (4.6%)	–
18.5–29.9	982 (81.4%)	241 (79.8%)	–
≥ 30	185 (15.3%)	47 (15.6%)	–
Comorbidities			
Number of comorbidities	1.0 (1.1)	0.9 (1.1)	0.417
Diabetes	133 (11.0%)	30 (9.9%)	0.584
Hypertension	518 (43.0%)	115 (38.1%)	0.125
High cholesterol	389 (32.3%)	93 (30.8%)	0.626
IHD	52 (4.3%)	16 (5.3%)	0.460
Stroke	17 (1.4%)	7 (2.3%)	0.259
Renal disease	22 (1.8%)	7 (2.3%)	0.576
Back pain	35 (2.9%)	7 (2.3%)	0.581
Depression	7 (0.6%)	2 (0.6%)	0.999
Surgical History			
Previous knee surgery	133 (11.0%)	37 (12.3%)	0.548
Previous hip surgery	236 (19.6%)	64 (21.2%)	0.527
Previous lumbar spine surgery	69 (5.7%)	28 (9.3%)	0.025*
Preop PROMs			
SF-36 PCS	27.4 (8.9)	26.7 (9.8)	0.233
SF-36 MCS	49.1 (12.1)	49.2 (12.2)	0.899
WOMAC	49.5 (20.9)	48.5 (20.9)	0.473
OHS	40.0 (9.2)	40.8 (9.3)	0.190
2-year PROM Improvement			
SF-36 PCS	+ 20.2 (12.0)	+ 19.8 (12.4)	0.601
SF-36 MCS	+ 6.9 (12.1)	+ 6.7 (12.6)	0.804
WOMAC	+ 41.4 (20.9)	+ 41.6 (22.1)	0.921
OHS	−24.1 (9.7)	−24.4 (10.2)	0.658
2-year Satisfaction			
Satisfied	69 (5.7%)	17 (5.6%)	0.951

Continuous outcomes are reported as mean (standard deviation) while categorical outcomes are presented as number (percentage)

*P*-values are calculated using two-sample *t*-tests for continuous variables and Chi-squared test/Fisher’s exact test for categorical variables

*: *P*-value < 0.05

### Patient satisfaction and PROM improvements

At 2 years postoperatively, 94.3% (*n* = 1422) of the patients were satisfied and 5.7% (*n* = 86) were dissatisfied (Table 2). Mean PROM improvements were + 20.2 (SD:12.1) for SF-36 PCS, + 6.9 (SD:12.2) for SF-36 MCS, + 41.5 (SD:21.1) for WOMAC and − 24.1 (SD:9.8) for the OHS.

### Model performance – patient satisfaction

**Fig. 1:**
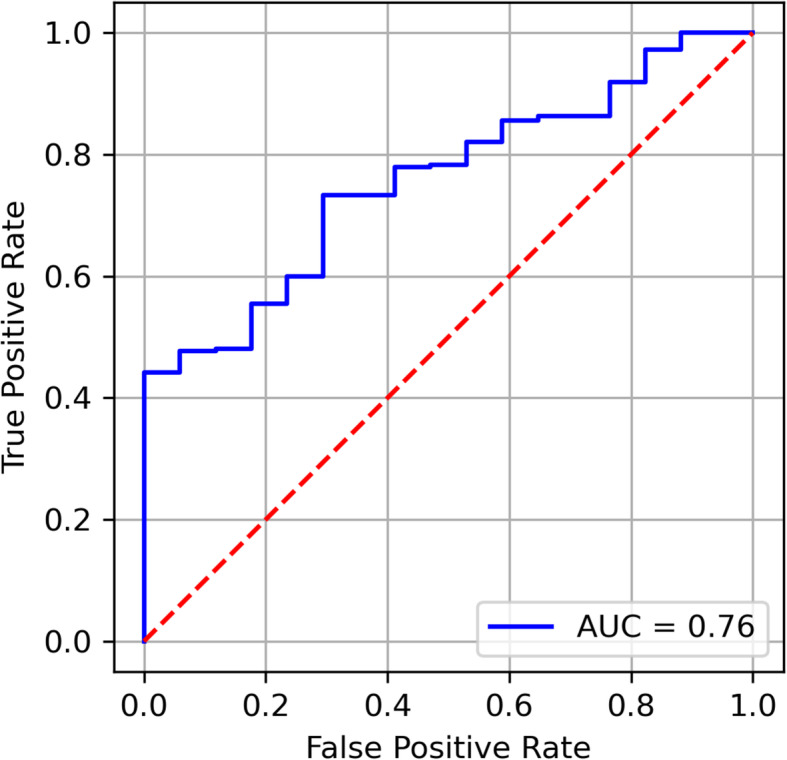
ROC curve for patient satisfaction using the LASSO model, achieving an AUC of 0.76

**Fig. 2:**
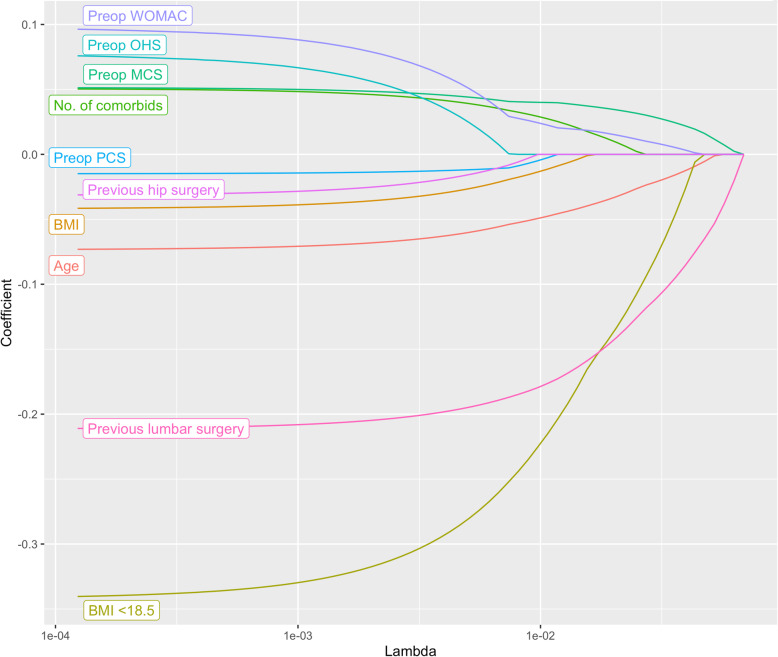
Regularization path for LASSO model

Evaluation results for predicting patient satisfaction are given in Table 3. Our ML models demonstrated fair discriminative ability in predicting patient satisfaction, with the LASSO model achieving a maximum AUC of 0.76 (Figs. 1 and 2). This was followed by SVM (AUC:0.74), RF (AUC:0.68) and XGB (AUC:0.66).

**Table 3:** Model performance for predicting patient satisfaction on test set (*n* = 302)

	LASSO	SVM	RF	XGB
AUC	0.76 (0.67–0.86)	0.74 (0.63–0.85)	0.68 (0.56–0.80)	0.66 (0.50–0.78)
Brier score	0.23	0.23	0.23	0.21
Threshold	0.50	0.52	0.53	0.58
Sensitivity	65.3%	57.9%	53.0%	50.5%
Specificity	82.4%	76.5%	76.5%	76.5%
Calibration slope	1.29	0.44	1.06	0.29
Calibration intercept	0.13	0.70	0.40	0.77

**Fig. 3:**
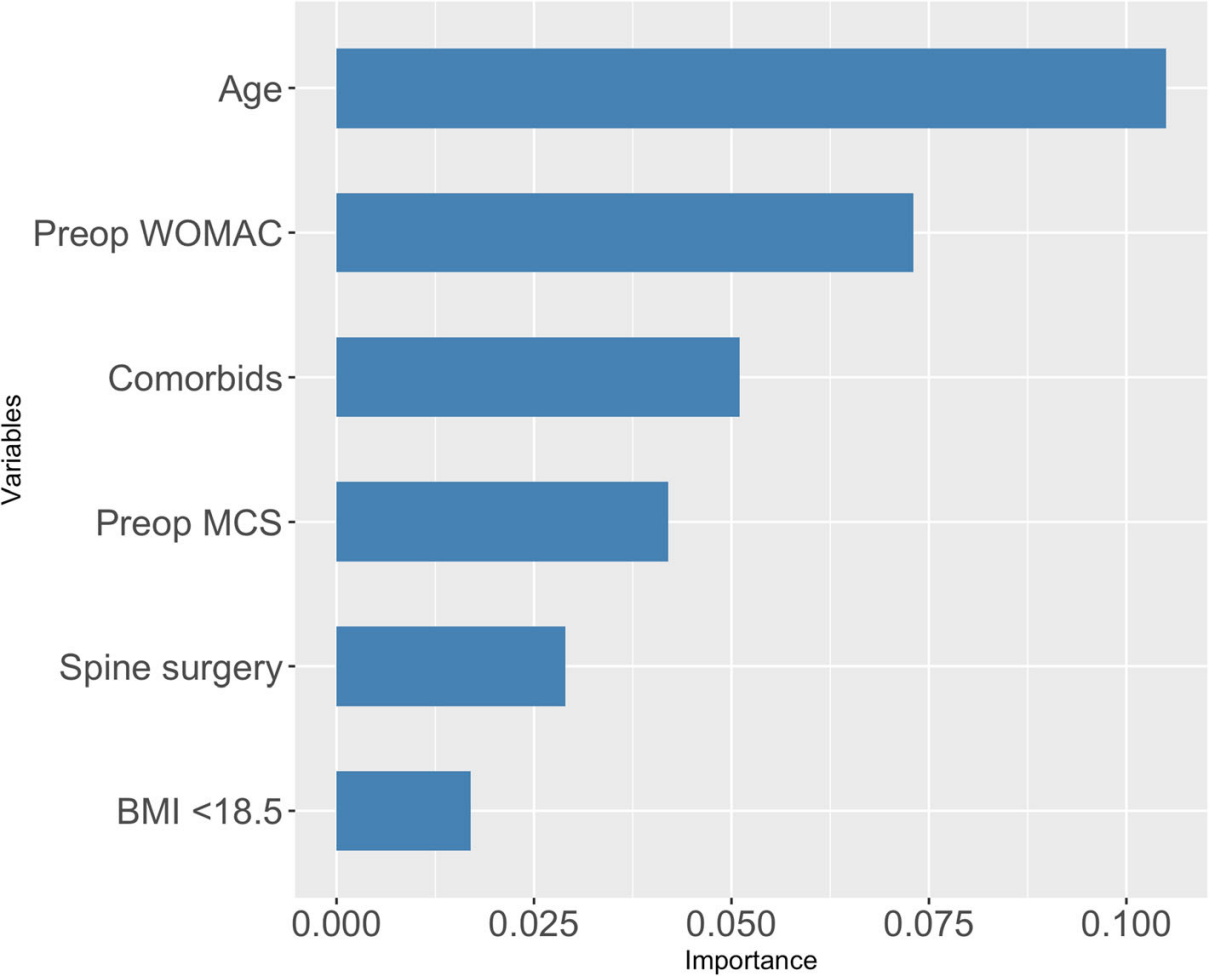
Most important predictors of patient satisfaction: (1) patient’s age, (2) preoperative WOMAC, (3) number of comorbidities, (4) preoperative MCS, (5) previous lumbar spine surgery and (6) low BMI (< 18.5)

Permutation importance (Fig. 3) revealed that the most important predictors of dissatisfaction in the preoperative model were (1) patient’s age, (2) preoperative WOMAC, (3) number of comorbidities, (4) preoperative MCS, (5) previous lumbar spine surgery and (6) low BMI (< 18.5).

## Discussion

Patient satisfaction is an important outcome measure in THA due to its ability to reflect subjective improvements in quality of life and the fulfillment of preoperative expectations [6, 29]. Our study was the first to use ML algorithms to predict patient satisfaction after THA. While prior studies by Kunze et al and Farooq et al have demonstrated the ability of ML algorithms to predict patient satisfaction after TKA, achieving an AUC of 0.77 and 0.81 respectively, their findings might not be generalizable to patients undergoing THA [21, 22]. Notably, several of the predictors identified in their studies (i.e. condylar-stabilizing implant, preservation of the posterior cruciate ligament, preoperative knee society score) were specific to TKA and not applicable for THA.

Our results have shown that ML algorithms using preoperative data had fairly good discriminative ability in predicting patient satisfaction after THA (AUC: 0.76). Although far from ideal, our results demonstrated the feasibility of using ML algorithms to identify high risk patients who may experience dissatisfaction after THA. This information could be used by surgeons to enhance preoperative counselling and manage patient expectations regarding THA, as prior studies have reported that interventions encouraging realistic expectations could improve satisfaction after joint arthroplasty [30]. Identification of at-risk patients may also allow for early intervention and optimization of their physical and mental health prior to THA, with prior studies showing that some predictors of satisfaction may be fully or partially modifiable [7, 31].

Our study also identified several modifiable and non-modifiable predictors of postoperative satisfaction. Some of our predictors, patient’s age (1) and number of comorbidities (3), were similarly observed in Kunze et al’s study amongst TKA patients [21]. Despite this, the impact of age on satisfaction remains controversial: While some studies have found that advanced age was associated with dissatisfaction, others have reported that younger patients are more likely to be dissatisfied, possibly due to their higher expectations and functional demands [6, 7]. Regardless, our current findings add to existing literature and reinforces the importance of these variables in a predictive model.

Next, we also identified preoperative WOMAC (2) and SF-36 MCS (4) as important predictors of dissatisfaction. This is consistent with prior literature which have reported an association between poorer preoperative physical and mental health state and dissatisfaction after TJA [5, 7, 29]. While the underlying mechanisms remain unclear, some authors have suggested that poorer preoperative PROMs may be indicative of poor musculoskeletal health, pathology in other joints (e.g., spine) and even increased pain sensitivity, all of which could potentially impede functional recovery and resolution of symptoms after THA [7, 32]. On the other hand, it is widely recognized that poor mental health influences a patient’s perception of disability and sensitivity to pain which may contribute to poorer satisfaction [33]. Recent studies have also suggested the possibility of mental health optimization, with Geng et al showing that psychological intervention in patients with depression could improve post-TKA satisfaction [31].

We also identified novel predictors of patient satisfaction, such as previous lumbar spine surgery (5). While there is an increasing understanding of the complex relationship between the spine and pelvis, with prior studies identifying lumbar spine pathology and surgery as risk factors for instability and dislocation after THA, much less is understood about how it influences patient satisfaction [34, 35]. Although our results suggest that previous lumbar surgery may be predictive of dissatisfaction, it should be noted that its impact is relatively small and further research is needed to better elucidate the underlying mechanisms. Lastly, we also observed that patients with low BMI (< 18.5) (6) were more likely to be dissatisfied. This could partially be due to poorer nutritional status being associated with poorer physical health, which in turn limits functional recovery and symptom resolution. Prior studies have also reported that patients with low BMI may experience more complications and a longer length of hospital stay after TJA [36, 37]. Although the importance of BMI was observed to be relatively low, it is highly modifiable and highlights the possibility of preoperative nutritional optimization.

Our study had several limitations. First, data used in this study were extracted from a single institution and thus it is unclear to what extent our findings can be extrapolated to other healthcare institutions. Although model performance in our study was assessed on an independent test set, future studies should focus on external validation of ML algorithms using data from other institutions. There was also a significant proportion (*n* = 488, 24.4%) of patients who were excluded from our study due to incomplete follow-up. This follow-up rate of 75.6%, while not ideal, is expected for studies with intermediate follow-up durations, with prior studies reporting similar follow-up rates of around 70–80% [32, 33]. Next, the performance of our ML models may have been limited by the input variables currently available in our joint replacement registry. Although our variables are largely similar to predictors identified in a systematic review by Gunaratne et al, there may be other factors such as the patient’s preoperative expectations, psychological coping mechanisms and other socio-economic factors which may influence postoperative satisfaction [7]. However, these factors are often overlooked by joint replacement registries and remain poorly understood, thus highlighting the need for further research in this area. Lastly, we acknowledge that our models may not be fully optimized in terms of calibration as the primary aim of our study was to demonstrate the ability of ML models to discriminate between the two outcomes.

## Conclusion

Machine learning (ML) algorithms demonstrated fair discriminative ability in predicting patient satisfaction after THA. We have identified modifiable and non-modifiable predictors of postoperative satisfaction which could enhance preoperative counselling and improve health optimization prior to THA.

## Data Availability

Data available on request.
